# High gain multi-band circularly polarized wearable leaky wave zipper MIMO antenna

**DOI:** 10.1016/j.heliyon.2024.e33024

**Published:** 2024-06-14

**Authors:** Tale Saeidi, Sahar Saleh, Sarmad Nozad Mahmood, Nick Timmons, Ahmed Jamal Abdullah Al-Gburi, Saeid Karamzadeh, Faroq Razzaz

**Affiliations:** a WiSAR Lab, Atlantic Technological University (ATU), Letterkenny, Co. Donegal, F92 FC93, Ireland; b Electrical and Electronics Engineering Department, Faculty of Engineering and Natural, Sciences, Istinye University, Istanbul, Turkey; c Department of Electronics and Communications Engineering, Faculty of Engineering, Aden University, Aden, 5243, Yemen; d Electronic and Control Engineering Techniques, Technical Engineering College, Northern Technical University, Kirkuk, Iraq; e Center for Telecommunication Research & Innovation (CeTRI), Fakulti Teknologi Kejuruteraan Elektrikal dan Elektronik (FTKEE), Universiti Teknikal Malaysia Melaka (UTeM), Malacca, 76100, Malaysia; f Millimeter Wave Technologies, Intelligent Wireless System, Silicon Austria Labs (SAL), 4040, Linz, Austria; g Electrical and Electronics Engineering Department, Faculty of Engineering and Natural Sciences, Bahçeşehir University, 34349, Istanbul, Turkey; h Electrical Engineering Department, College of Engineering, Prince Sattam Bin Abdulaziz University, Al-Kharj, 16278, Saudi Arabia; i Faculty of Engineering and Information Technology, Taiz University, Taiz, 6803, Yemen

**Keywords:** 5G, Sub-6 GHz, Zipper antenna, Wearable leaky wave antenna, Multi-band antenna, WBAN

## Abstract

A miniaturized, multi-band, four-port wearable Multiple Input Multiple Output (MIMO) antenna is proposed, which contains a leaky wave textile antenna (LWTA) on denim (ε_r_ = 1.6, tanδ = 0.006) as substrate and Shieldit Super Fabric as conductor textile. The concept in this work involves incorporating the metal and plastic zipper into the garment to function as an antenna worn on the body. Simulations and measurements have been conducted to explore this idea. The LWTA has dimensions of 40 × 30 × 1 mm³. Every two ports are separated by a zipper with two different kinds of materials: Acetal Polymer Plastic (APP) and 90 % brass to improve the isolation, gain, and Impedance bandwidth. The antenna operates in the frequency ranges covering the L, C, S, and X bands. Additionally, diversity performance is evaluated using the Envelope Correlation Coefficient (ECC) and diversity gain (DG). Simulation and measurement findings agree well, with a maximum gain of 12.15 dBi, low Specific Absorption Rate (SAR) based on the standards, DG greater than 9.65 dB, circular polarization (CP), and strong isolation (<-23 dB) between each port. Since the antenna's characteristics do not change significantly under bending and when the zipper is opened, the proposed antenna is a viable candidate for body-centric wireless communications on the battlefield. For example, it can facilitate communication covering wireless local area network (WLAN) and fifth-generation (5G) communications.

## 1 Introduction

Wireless communications leverage MIMO tech for speed and reliability, using multiple antennas at both ends. Polarization diversity reduces spatial constraints, mitigating multipath fading. Therefore, techniques like defected ground structure (DGS), split ring resonators (SRRs), and electromagnetic band gaps (EBG) maintain performance in MIMO systems. Wireless Body Area Networks (WBANs), crucial for identity systems and healthcare, demand flexible materials but face signal disruptions due to movement, necessitating diverse setups. Hence, compact wearable MIMO systems that benefit from vertical polarization can offer promising outcomes but pose setup challenges. Multi-band antennas are essential for accommodating various standards, like vertical polarization, which aids in on-body propagation in WBANs. Furthermore, wearable systems require physical elasticity, robustness, and connectivity during motion [[1], [2], [3]]. To face the above challenges in wearable antennas, researchers have investigated various types of materials and antennas, such as conductive textile antennas [4], elastic substrate-based antennas [5], and button-like antennas [6]. Most of the on-body antennas are solely made on textile substrates. However, the body's textiles can bend and stretch, thereby altering the resonance frequency, bandwidth, and radiation efficiency of textile antennas. To overcome this issue, garment components such as buttons were incorporated instead of fully textile substrates [[7], [8], [9]].

Other than buttons, zippers, as a common part of any garment, were also utilized as an antenna of a part of a wearable antenna. In research, the direction of the zipper was altered by moving the zipper handle up and down. As a result, the radiation pattern was naturally reconfigurable towards the zipper. The dimensions of 100 × 70 mm^2^ offered two frequencies of 2.4 GHz and 5.5 GHz [10]. In addition, the metal zipper of a purse was presented as an off-body antenna, and prototypes and measurements were performed. A fractional bandwidth of 4.92 % was reached at 2.44 GHz of the industrial, scientific, and medical (ISM) bands, with a gain of around five dBi considering all conditions of zipper-like close and opened [11]. In a separate study, a belt antenna featuring a ground plane made from textile materials incorporating an EBG was developed. This particular belt antenna operates within the 2.45 GHz frequency range utilized in ISM bands, specifically for Bluetooth low-energy communications. A textile ground plane was devised to be integrated into the trouser fabric behind the belt. This integration isolated the antenna from the body while enhancing its radiation characteristics. The antenna achieved a maximum realized gain of 7.94 dBi and a minimum SAR of 0.04 W/kg at an input power of 0.5 W, with dimensions of 90 × 12 mm^2^ [12].

Apart from the challenges that wearable antennas face and mentioned before, having high data rate transition, circular/dual polarization, multipath fading, and path loss should also be considered. Therefore, a MIMO antenna designed on a textile utilizing garment components such as zippers and buttons will be promising to fulfill most requirements for a highly diverse wearable MIMO antenna. This integration allows the antenna to be seamlessly integrated into the fabric, easily connected and disconnected, resistant to degradation caused by folding, and has sound isolation from the body. The antenna should also cover various communication bands while offering dual-polarization, pattern diversity, and miniaturization since these features enhance internet connections, eliminate polarization inconsistencies, and improve robustness towards bending. However, achieving these requirements simultaneously in a wearable MIMO antenna with a zipper has not been accomplished yet. Even those presented in Refs. [[10], [11], [12]] overlooked most of these requirements. Hence, a circularly polarized or polarization-diverse (dual or triple polarization) cloth MIMO antenna incorporating a zipper can be employed to face most of these issues.

Various MIMO antennas were designed on both flexible and rigid substrates. However, the most recent MIMO antennas with four-ports will be considered for comparison with the proposed antenna later on [13,14]. presented two transparent 4-port MIMO antenna with the dimensions of 45 × 55 mm^2^ and 66 × 45 mm^2^, respectively. They were constructed using PET and silver oxide, and flexible Melinex substrate and could achieve peak gain of 2.49 and 0.53 dBi, with minimum efficiency of 40 % and 41 %, respectively. They also offered satisfactory values for diversity gain (DG) and error correction coding (ECC). A compact wearable antenna system utilizing MIMO technology was proposed for the new radio band 79 (4.4–5 GHz). It consisted of four coplanar waveguide-fed antennas, each hexagonal-shaped and printed on a polyimide substrate (88.7 × 68.7 mm^2^). With a focus on enhanced isolation, it achieved isolation levels exceeding 17 dB and peak gain ranging from approximately 3.8 to 4 dBi across the frequency range. The antenna also showed a radiation efficiency of 88 %–90 %. Utilizing a flexible substrate allowed it to conform to different shapes, validated through bending analysis [15]. Crafted for operation within the Ultra-Wideband (UWB) spectrum (3.1–12 GHz), the antenna obtained an impressive impedance bandwidth of 8.9 GHz. The unit cell and MIMO antenna dimensions were 25 × 20 mm^2^ and 51 × 51 mm^2^, respectively. It demonstrated notable performance with maximum efficiency at 93 % and a peak gain of 4.62 dBi. Diversity metrics revealed ECC values below 0.08 and DG values below 9.99 dB. Additionally, CCL and Total active reflection coefficient (TARC) values remained under <0.13 bits/s/Hz and <−12 dB, respectively [16]. Using jeans textile material, a compact MIMO antenna with dual or quad elements, was proposed for Worldwide Interoperability for Microwave Access (WiMAX) and 5G applications. The wideband characteristics of a single-element antenna covering a frequency range from 2.74 to 4.41 GHz (47 %) were analyzed. Excellent isolation exceeding 19 dB and maximum gain below five dBi were achieved across the entire application band with dimensions of 58 × 55 mm^2^. Diversity parameters such as ECC <0.2, DG > 9.6, and mean effective gain (MEG = ±0.3 dB) were observed to fall within satisfactory ranges [17]. However, it is noteworthy that the antenna's gain was relatively low, and not all aspects of diversity investigations were thoroughly examined in the recent previous works explained above.

The realization of an interconnected and intelligent environment for everything and everyone is becoming increasingly feasible, promising a significant market opportunity in the near future. While existing wearable and body-centric antennas have demonstrated commendable performance, particularly for specific uses, many require additional components to be added to the body or equipment, and there is still room for improvement in performance across diverse applications. However, it's crucial to consider hurdles wearable MIMO antennas encounter, such as high data rate transmission, circular/dual polarization, multipath fading, and path loss. Therefore, exploring new antenna designs holds practical value. Utilizing the metal/plastic zipper already integrated into clothing as an antenna or incorporating it as part of an antenna to enhance its characteristics presents a promising concept. Still, many of these essential requirements have been overlooked. One-port, two-port, and three-port wearable MIMO button antennas offered by Refs. [[7], [8], [9]] yielded acceptable results as single-terminal and MIMO antennas. However, they exhibited several disadvantages. For instance, the design procedure did not consider circular polarization, high data rate transition, broad bandwidth, and miniaturization [7]. Both antennas in Refs. [7,8] were fed from behind by an SMA port, which poses challenges in real-world scenarios in the field. Two dual-band antennas presented in Refs. [8,9] had high gain and wide bandwidth. However, since they could only cover two bands, they are still limited to real-world experiences like battlefields, where broader coverage is necessary. More frequency band coverage and wider bandwidth are required because the wearable antenna might bend in harsh environments, causing some frequency bands to shift accordingly. Besides, these buttons were prone to movement since they were connected by single wires, unlike zippers that can be sewn to the clothes. Therefore, a zipper is preferred over a button, and considering more than three ports (four ports) fed from the side, it is chosen for the proposed antenna to enhance the BW and data bit rate transmission.

This study proposes a unique multi-band zipper antenna incorporating a four-ports wearable MIMO LWA that enables lower 5G band and sub-6 GHz communications (also covering L, C, S, and X bands). LWA was selected for this work owing to its ability to do beam steering and frequency scanning, which is suitable for body-centric and WBAN applications on the battlefield. Multi-band capability, compactness, and user comfort are improved by including metal and plastic zippers as a standard part of a garment. Unlike commonly used cloth parts, such as buttons, they are not as sensitive to bending and harsh environments. The zippers are made of two different materials: 91 % brass and Acetal Polymer Plastic (APP), the same as actual zippers in the market. This approach aims to be more practical and replicate the same scenario as when genuine zippers in the market are used. The antenna consists of a novel leaky-wave textile antenna (LWTA) with a completed ground on the first layer and a neutralization layer on the second layer. The novel design of LWTA offers a broadside and wide radiation pattern, high gain, and radiation efficiency. It achieves high isolation by employing nine nested L-shape slots on one side combined with a broader L-shape slot on the other side, separated by a T-shape slot. The antenna can transmit a circular broadside radiation pattern and offer a monopole-like resonant mode, which generates an omnidirectional radiation pattern in the reduced active region. This monopole-like radiation pattern is utilized in the WiMAX and lower 5G spectrums for on-body data transfer. It can also offer an acceptable SAR level in all working bands less than the European standard's cut-off values.

After an informative introduction in Section 1, section 2 focuses on evaluating and establishing the conceptual framework of the proposed zipper MIMO antenna, designed explicitly for WBAN applications and 5G communications. Section 3 thoroughly investigates the antenna's modeling and presents experimental results. Finally, Section 4 concludes by summarizing the essential findings and providing recommendations.

## 2 Antenna geometry

This section outlines the antenna configuration and design procedures. The MIMO LWTA is initially constructed for specific frequency bands before integrating the zipper. The radiation characteristics of the two-port configuration are examined and extended to a four-port setup. Incorporating the zipper enhances isolation between ports, improving performance. Two neutralization techniques—nested complementary rectangular split ring resonator (CRSRR) cells and S-shaped resonators—are employed to enhance isolation, impedance bandwidth, radiation efficiency, bandwidth, and gain while suppressing surface waves. The antenna mitigates omnidirectional backside radiation with transversal and longitudinal slots in T and L shapes, achieving a broadside radiation pattern. Integration of zippers and a neutralization network yields constructive high broadside radiation characteristics. Current and electric-field concentrations were analyzed, and then closed and open zipper configurations were assessed at various angles to elucidate antenna operation. Afterward, mobility and optimization studies are conducted using nonuniform and measurement phantoms and actual human subjects, focusing on chest, arm, and limb regions for authentic conditions simulation. Critical parameters are optimized based on actual values obtained from principle equations before detailing the design process.

### 2.1 Design of proposed MIMO LWTA without the zippers

**Fig. 1:**
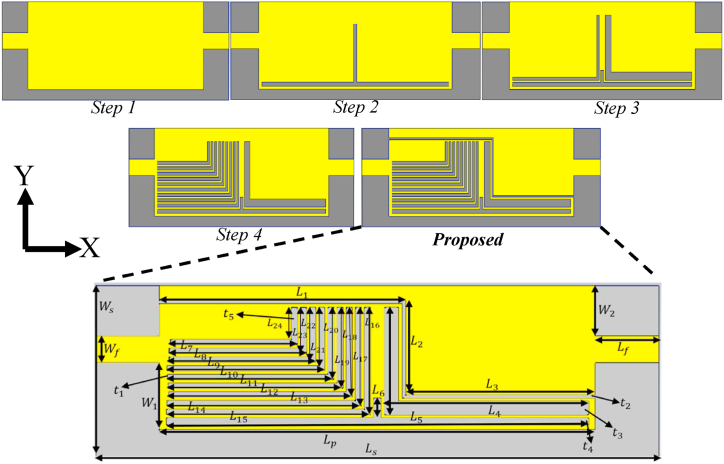
The design configuration of a single antenna, including each step of the design and the proposed prototype.

Fig. 1 illustrates various views of the proposed MIMO LWTA. Each design stage starts from two ports and progresses to four ports, both separately and integrated with the neutralization networks later. Two zippers with different materials, namely, APP and brass, are utilized to account for real-world scenarios. Since a long zipper on a shirt may alter the antenna's radiation characteristics, only a small zipper is considered in the simulation. In real-world scenarios, the small zipper is typically used for pockets on the chest or shoulder of the shirt. The effects on the human body will be discussed later in the results section of the article.

The proposed LWTA incorporates a transmission line-fed rectangular area, with one end of the antenna serving as an excitation point and the other end terminated with a 50-Ω load (refer to Fig. 1, step 1). Initially, fundamental equations, as provided by Ref. [18], are employed to determine the dimensions of the rectangular patch and transmission line, enabling the antenna's design at 3.2 GHz (where λ_0_/2 < 46 mm). However, these equations, being well-established principles, are not reiterated here. Subsequently, the design process for the proposed LWTA begins, adhering to the following rules, equations, and standards, which are essential for every LWA. It is worth noting that altering the length of an antenna can significantly affect its resonant frequency. Typically, an antenna's resonant frequency is inversely proportional to its length; as the antenna's length increases, its resonant frequency decreases, and vice versa. The principles of electromagnetic waves and the specific construction of the antenna govern this relationship. The design of the leaky wave structure prioritizes high directivity and gain, low loss, and structural simplicity. LWAs belong to a class of traveling-wave antennas characterized by a wave propagating along a long structure compared to the wavelength. They generate currents that propagate along its longitudinal direction, resulting in end-fire and back-fire radiation patterns due to the open-stopband (OSB) phenomenon, as described in Refs. [19,20]. These antennas can be effectively modeled using balanced Composite right-/left-handed (CRLH) transmission lines, which combine the effects of capacitors and inductors. Enhancements are necessary to enable outward radiation from the body in a broadside direction.

**Fig. 2:**
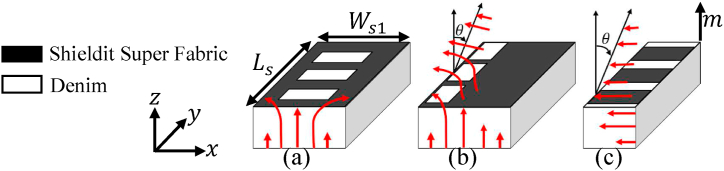
Asymmetry radiation principle: (a) ${\text{TE}}_{10}$ mode in even structure, (b) leaky ${\text{TE}}_{10}$ mode in uneven structure, and (c) leaky ${\text{TE}}_{01}$ mode in even structure (θ is the approximated maximum angle of the radiation).

The proposed LWTA incorporates asymmetric elements, including longitudinal slots aimed at improving the OSB and transverse slots designed to eliminate OSB issues, functioning like a series of left-handed capacitances (CL). Consequently, these elements modify the radiation pattern, making it more effective in the broadside direction rather than end-fire or back-fire. Moreover, these slots serve to enhance isolation between antennas within the array and mitigate surface waves generated by LWAs. The characteristic mode method is employed to analyze and design these slots and evaluate their impact on the radiation pattern from the initial stage to the final stage of the design process, considering the principles of asymmetry radiation as depicted in Fig. 2 [21].

The periodic slots do not cover the entire substrate width ($W_{s1}$) and are positioned at an offset from one side (refer to Fig. 1, Fig. 2). Additionally, the waveguide port must be excited with the ${\text{TE}}_{10}$ mode, rather than the TE sub 01 mode as in the dielectric inset. Following the proposed shape of the antenna and the improvement of the OSB issue through asymmetrical structure, the radiation mechanism of the new antenna is based on the asymmetry principle shown in Fig. 2. When the periodic slots are symmetrically located concerning the side walls, the vertically polarized ${\text{TE}}_{10}$ mode does not induce any horizontal field in the antenna aperture, as depicted in Fig. 2(a). This principle is utilized by the transverse parts of the T and L-shaped slots in the proposed design. It implies that the electromagnetic waves emitted from the port exhibit polarizations perpendicular to the longitudinal planes of both the antenna and the transmission line. Considering the stub-loaded LWA, the parallel plates (the patch and ground layers) function as a filtering mechanism, allowing only the horizontal field to reach the waveguide aperture, located at the height of *m* from the dielectric interface in the space. Therefore, the symmetrical periodic structure renders the ${\text{TE}}_{10}$ mode nonradiative when m is sufficiently high. TThe slots must be asymmetrically positioned to induce a potential difference in the parallel plates This difference of potential generates a horizontally polarized TEM wave, which can propagate to the aperture and radiate into the free space region, as depicted in Fig. 2(b). The longitudinal parts of the T and L-shaped slots in the proposed design operate based on this principle. Thus, by adjusting the level of slot asymmetry, one can control the excitation level of this radiating horizontal wave, thereby aalteringthe leakage rate of the ${\text{TE}}_{10}$ leaky mode. In contrast, the dielectric inset operates based on the excitation of the ${\text{TE}}_{10}$ mode, which is horizontally polarized itself. The ${\text{TE}}_{10}$ mode is solely responsible for the antenna's radiation; therefore, there is no need to introduce any asymmetry to induce radiation in the structure, as illustrated in Fig. 2(c). Moreover, the only way to control the inset's leakage rate is by altering the slot aperture. However, in this novel design and combination of slots, the leakage rate can be controlled by adjusting the slot asymmetry (the spacing between the slots and the periodic scale of nested L-shaped slots) while maintaining the inset space value unchanged.

The antenna at stage 1 utilizes simple microstrip lines on a dielectric substrate layer, behaving similarly to a transmission line where the wave propagates and leaks upon encountering a discontinuity in the structure. Additionally, the wave's energy traveling on the surface can be radiated away toward the other port. Similarly, the currents from the source propagate down the microstrip line and are scattered by the surface-modulated impedance to produce the desired radiation pattern. Resonance is achieved at 3.2 GHz, along with another resonance at a higher band around 7.6 GHz. In stage 2, a T-shape slot is cut from the rectangular patch to create discontinuity and leakage. The loss created by the leakage at the T-shape slot interrupts the current flow over the patch, enhancing the array's impedance bandwidth and beamwidth. This also creates another resonance around 5.6 GHz and widens the higher band around 7.6 GHz. However, the working bands at 3.2 GHz, 5.6 GHz, and 7.6 GHz are shifted towards higher bands, thus requiring a reduction in the length of the transverse slot of the T-shape slot to compensate for it. The transverse part of the T-shape slot behaves as a series of left-handed capacitance, affecting the radiation pattern so that it is not directed towards the end-fire and back-fire. In stage 3, two L-shape slots with different thicknesses are added on both sides of the T-shape slot. The L-shape and T-shape slots can produce a multiband structure, with two more resonances created around 4.25 GHz and 8.65 GHz. The first resonance is widened, and the third one is shifted slightly to a lower band. At stage 4, the L-shape slot on the left side of the T-shape slot is converted into a periodic structure of nine L-shape slots. This periodic structure introduces more resonances and widens the bandwidth at the working bands. Finally, the two ports are separated by cutting an S-shape slot with a total length of ($L_{1}$ + $L_{2}$ + $L_{3}$) to enhance the isolation between ports one and two. After using equations and analyzing the LWTA's characteristic mode, the actual values of the antenna parameters are obtained and optimized. The optimized values for each part of the antenna are indicated in Table 1.

**Table 1:** The structural parameter values of the proposed antenna.

Parameters	Values (mm)	Parameters	Values (mm)	Parameters	Values (mm)	Parameters	Values (mm)
$L_{s}$	40	$L_{4}$	14.50	$L_{10}$	11.25	$L_{16}$	8
$L_{p}$	31	$L_{5}$	30	$L_{11}$	11.8	$L_{17}$	7.35
$L_{f}$	4.50	$L_{6}$	1.5	$L_{12}$	12.5	$L_{18}$	6.70
$L_{1}$	18.00	$L_{7}$	9	$L_{13}$	13	$L_{19}$	6
$L_{2}$	7.50	$L_{8}$	9.75	$L_{14}$	13.8	$L_{20}$	5.40
$L_{3}$	13.00	$L_{9}$	10.50	$L_{15}$	14.50	$L_{21}$	4.35
$L_{22}$	3.70	$L_{m1}$	29.00	$W_{m1}$	35.00	$W_{s1}$	30.00
$L_{23}$	3.00	$L_{m2}$	24.00	$W_{m2}$	30.00	$t_{1}$	0.50
$L_{24}$	2.40	$L_{m3}$	19.00	$W_{m3}$	25.00	$t_{2}$	0.20
$L_{f1}$	10	$L_{m4}$	14.00	$W_{m4}$	20.00	$t_{3}$	1.00
$L_{s1}$	40	$L_{m5}$	10.00	$W_{m5}$	16.00	$t_{4}$	0.50
$r_{1}$	0.50	$S_{3}$	8.00	$W_{f}$	2.00	$W_{2}$	5.00
$S_{1}$	3.50	$S_{4}$	8.00	$W_{1}$	3.50	$W_{s}$	15.00
$S_{2}$	5.75	$S_{5}$	8.50	$W_{3}$	4.38		

**Fig. 3:**
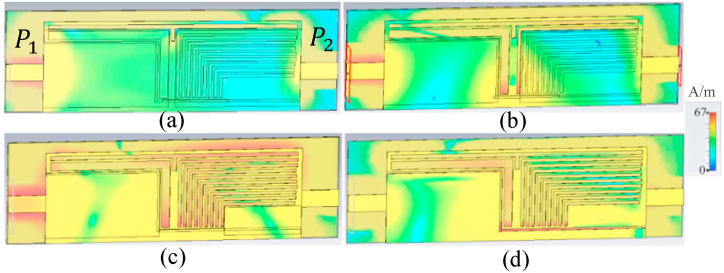
The SCD of the proposed antenna (a: stage 2, b: stage 3, c: stage 4, and d: final).

Following the analysis of the proposed design and the design steps illustrated in Fig. 1, the surface current distribution (SCD) of the proposed antenna is presented in Fig. 3 for each step of the design. As depicted in Fig. 3, the current density of the antenna is strongest at step 2 of the design. Additionally, the SCD exhibits the highest level around the T-shape slot and the two L-shape slots on both sides of it. This trend is consistent with the SCD for the other two design steps shown in Fig. 3(c) and (d), where the SCD shows the highest level around the periodic L-shape slots and the S-shape slot.

**Fig. 4:**
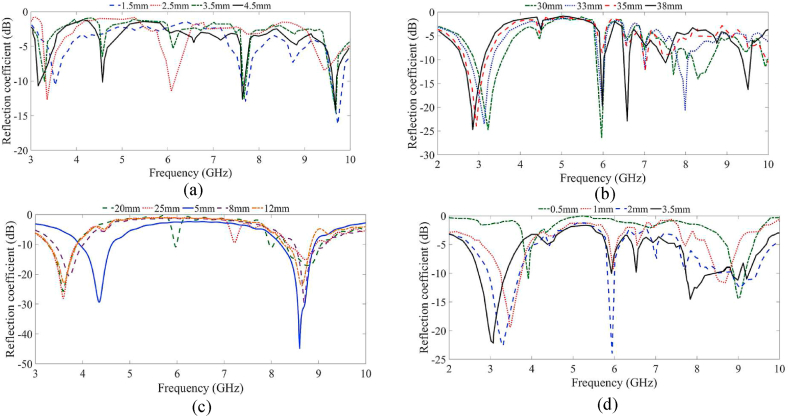
Parametric study of the proposed antenna on (a) $L_{f}$, (b) $L_{p}$, (c) $L_{5}$, and (d) $W_{f}$.

The antenna can be tuned and optimized through a parametric study, where four of the most influential parameters affecting the initial design steps are adjusted. Fig. 4 illustrates the variations in the feed line length, the length of the patch, the T-shape slot length, and the width of the feed line. Increasing the feed line length (Fig. 4(a)) shifts the operating band to lower frequencies, while decreasing it shifts the band to higher frequencies. The width of the feed line (Fig. 4(d)) impacts matching and can widen the bandwidth at the resonant frequency during the design process, but resonance can be dramatically shifted to a higher band if the width is too low. The patch length also affects resonance, turning it to lower or higher bands and creating additional resonances when intersecting with other slots. For example, a patch length of 38 mm shifts the first resonance to a lower band, creating undesired additional resonances. Similarly, the length of the T-slot affects the first resonance and other poles in the band. Since the other parameters do not significantly affect the antenna impedance bandwidth, they are not discussed in detail here. After briefly investigating the parametric study without the zippers, the antenna's characteristics are evaluated by integrating zippers and extending the antenna to a four-port configuration to increase the bandwidth and facilitate data transmission in real scenarios.

### 2.2 Proposed MIMO LWTA integrated with the zippers

**Fig. 5:**
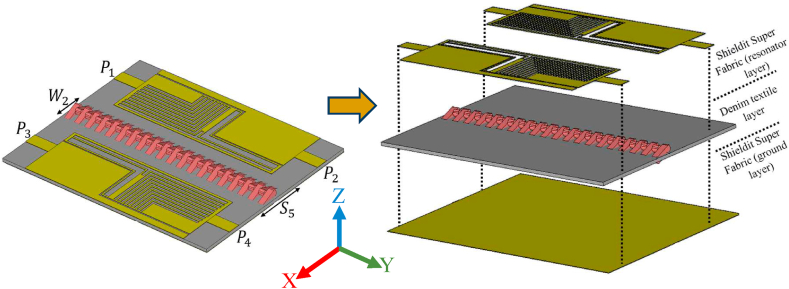
The integration of the proposed four-port MIMO LWTA integrated with zippers.

To enhance the radiation characteristics of the antenna, including bandwidth, gain, and directivity, and to enable broadside directional capabilities of the LWA, the investigation includes the addition of zippers made of APP and brass combined with different materials, as shown in Fig. 5. These brass zippers are connected to the entire ground layer of the LWA, resulting in lower Specific Absorption Rate (SAR) values compared to a configuration with a defective ground. The grounded brass zipper acts as shunt left-handed inductors, combined with the transverse slots of the leaky wave, suppressing the Open-Stopband (OSB) issue of the LWA and enhancing the directivity and gain towards broadside radiation. Additionally, the zipper made of APP functions as a dielectric resonator, similar to how dielectric resonator antennas (DRAs) are conventionally employed for high-frequency purposes, achieving a broad impedance bandwidth and adequate gain. DRAs also reduce surface waves and minimize conductor losses at higher frequencies [22]. Consequently, the APP zipper enhances the directive gain and radiation efficiency. This configuration provides various advantages, including expanded bandwidth, decreased spurious radiation, and the ability to operate in multiple frequency bands. These advantages become evident when examining the changes in the reflection coefficient resulting from incorporating zippers around the LWA. Before delving into the explanation of neutralization networks, it's essential to understand how surface currents and electric fields work around the design.

**Fig. 6:**
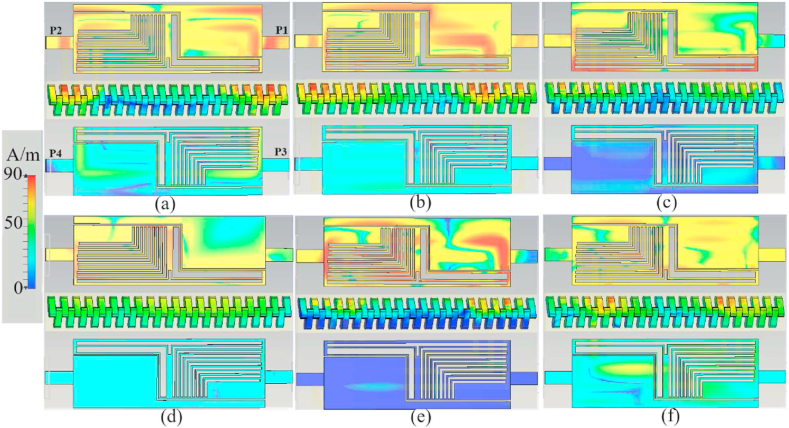
The SCD of the four ports LWTA at a: 3.4 GHz, b: 4.2 GHz, c: 5.6 GHz, d: 7.25 GHz, e: 8.5 GHz, and f: 9 GHz.

Fig. 6 illustrates the SCD in MIMO LWAs with four terminals, offering insight into the propagation of waves from Port 1 (P1) through the slots and to the other ports. The dynamic range of the SCD is represented by red, which indicates the highest density at 90 A/m, and blue, which indicates nearly zero density. Fig. 6 reveals that significant currents are present at higher frequencies, such as 7.25 GHz, 8.5 GHz, and 9 GHz, around the nested L-shaped slots and the T-shaped slot. Conversely, stronger currents are observed around the transmission lines and the patch at frequencies lower than 5.6 GHz. At 7.25 GHz, the current density is notably low when the current is distant from the slots due to the change in current direction caused by the addition of the following order of the L-shaped slot. Significant current density is also observed in the nested L-shaped slots, the T-slot, and the patch. This significant current density extends to other antenna parts at 9 GHz.

**Fig. 7:**
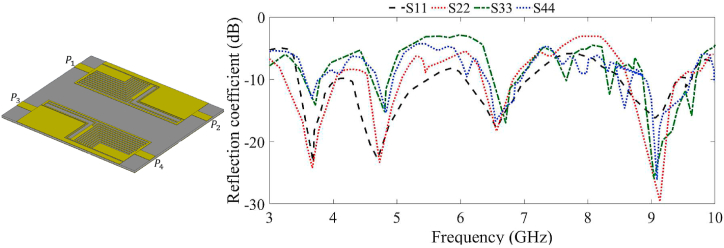
The simulated reflection coefficient results of the proposed four-port MIMO antenna without zipper integration.

Fig. 7 illustrates the reflection coefficient results of the four-port MIMO LWTA. It also depicts that the MIMO LWTA exhibits that resonance around 3.4 GHz becomes more expansive, and the resonance around 5.6 GHz is suppressed due to the mutual coupling effect that occurs after extending the antenna to four ports. Furthermore, it is essential to note that in this case, the LWTA does not include the neutralization network and the zippers, and it features a complete layer of ground (suppressing the band from 5 GHz to 6.5 GHz and reducing the radiation efficiency). Consequently, activating the other ports creates additional resonances at higher frequency bands, which cause the resonances to shift to higher and lower frequencies. However, the assessment of the MIMO antenna's radiation efficiency, gain, and other desired characteristics is yet to be discussed. Therefore, including loadings and other design components is necessary to improve the antenna characteristics and meet the requirements for a multiband MIMO antenna.

### 2.3 Integration of neutralization networks with the MIMO LWTA

**Fig. 8:**
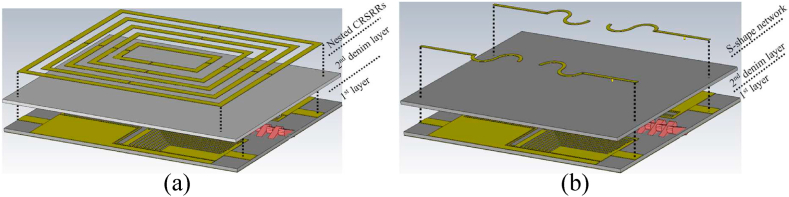
The simulated prototypes of the naturalization networks, a: nested CRSRRs network and b: the S-shape network.

Despite the improvement after adding a zipper to the antenna, the isolation between the ports is yet to be considered acceptable since it's around −14 dB. Moreover, the existing spacing between each pair of ports in the LWTA has already slightly reduced isolation, as miniaturization has been one of the main objectives throughout the design process. Different methods are available to enhance MIMO antennas' isolation capability, commonly called decoupling techniques. Consequently, these methods should be combined to enhance the antenna dimensions and improve performance. As previously mentioned, zippers feature two distinct materials (similar to the current zippers on the market). The selection of neutralization networks was tailored to enhance their effectiveness and to be more realistic in the real world (Fig. 8).

The arrangement of five nested RSRRs produces four complementary RSRRs that possess characteristics similar to those of the CRSRRs, thereby enhancing the radiation performance of the LWA. Unlike the SRRs, the CSRRs exhibit a significantly lower resonant frequency for the same physical dimensions. As a result, they can be designed to resonate at lower frequencies. Consequently, their integration with the LWA enables a shift towards lower frequency bands while preserving the passbands and resonances established by the LWA itself, given that a CRSRR exhibits dual-band characteristics. In this work, five RSRRs or four CRSRRs are involved. The nested structure of CRSRRs allows for maintaining a quasi-static resonance frequency below the regular resonance frequency, resulting in more compact devices. This highlights the benefits of the resonance properties of anisotropic particles compared to other techniques (it will be compared with similar works later in Table 3). Furthermore, any individual part of the zipper's teeth can create a non-uniform field pattern, displaying a pronounced magnetic or electric response. However, a relatively weak coupling between neighboring teeth indicates the quasi-static nature of effective parameters. Consequently, integrating the CRSRR and the brass zipper leads to a degradation of the strong electromagnetic fields that could generate a high-quality factor, resulting in narrower bandwidths at desired frequency bands. As a result, the operational bandwidth around the desired resonances increased, enhancing the antenna system's radiation characteristics, including directive gain, radiation efficiency, and isolation.

Initially, a brass zipper is employed, followed by integrating the second layer, which incorporates nested CRSRR cells, with the antenna. To investigate the operational principles of CRSRRs, an external magnetic field is applied along the Z-axis; hence, the electromagnetic force (EMF) is detected at each CRSRR. The EMF is generated through the connection between two rectangular metal rings, resulting in a current flow between them facilitated by the capacitance formed within the interior gaps of the rings. The CRSRRs can be represented by an equivalent circuit of a parallel LC resonator, where a current must traverse from one ring to the other to demonstrate the overall inductance. The capacitances associated with the CRSRR structure, including the two halves and the rings, and the capacitances related to the split rings' gaps are considered.

**Fig. 9:**
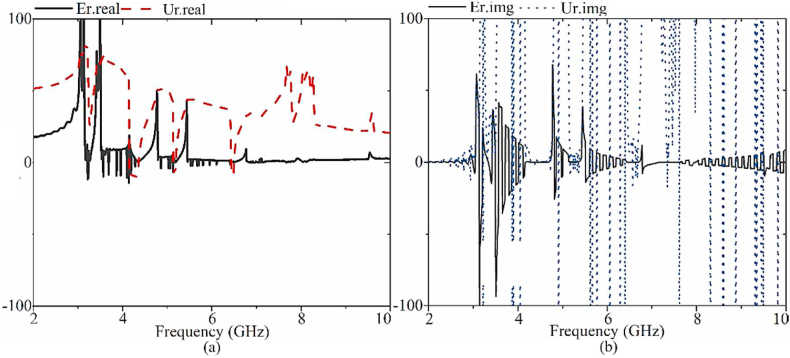
The extracted permittivity and permeability results of the CRSRR.

In Fig. 9, the results of the retrieved permittivity ($\varepsilon_{r}$) and permeability ($\mu_{r}$) (both real and imaginary parts) of the CRSRR unit cell are displayed. Each unit was meticulously designed to correspond to a particular frequency. The five CRSRRs are arranged in a specific sequence based on their frequencies. The CRSRR unit cell forms four pairs of CRSRRs, each exhibiting different resonance frequencies ($f_{r}$) of 3.1 GHz, 3.4 GHz, 5.6 GHz, and 7.2 GHz. The CRSRR design procedure was simulated using a virtual microwave studio (CST MWS) to ensure accuracy. Initially, the CRSRR structure was positioned close to the power line. The smaller CRSRRs showed resonances in the frequency range of 5.55–5.65 GHz and 6.85–7.15 GHz, while the larger CRSRRs exhibited resonances in the range of 3.10–3.3, 3.45–3.55 GHz. The obtained −2 dB transmission coefficients present a high signal-to-noise ratio, signifying excellent performance. Moreover, the desirable frequencies exhibit a nearly negligible refractive index. They perform a similar function to a photonic band gap, attenuating undesired resonances while enhancing the desired ones. The spacing between the CRSRRs is fine-tuned post-design to optimize the impedance bandwidth and maintain it within an acceptable range for reflection and transmission coefficients. Additionally, they hinder surface waves and unwanted fringing fields near the antenna's edges and ports.

**Fig. 10:**
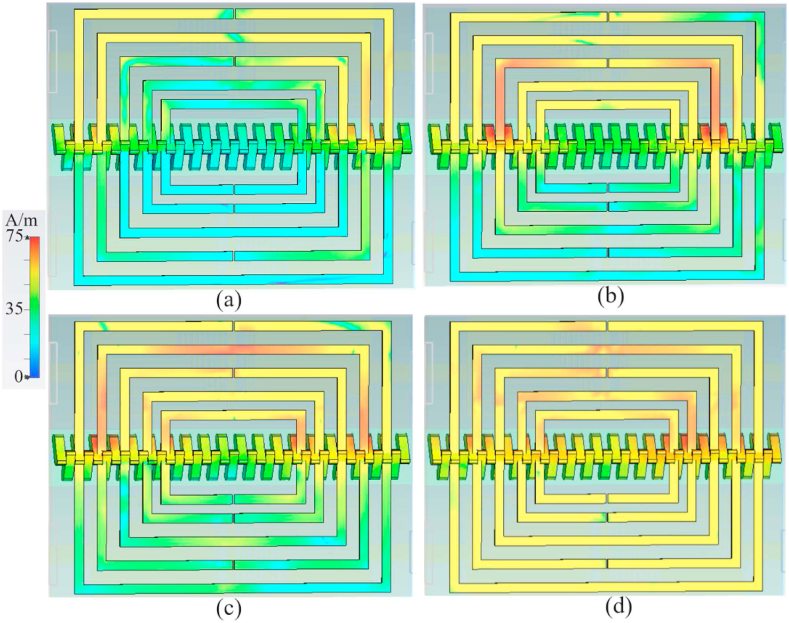
The SCD at a: 3.4 GHz, b: 5.6 GHz, c: 7.2 GHz, d: 8.2 GHz.

Fig. 10 illustrates the SCDs in the CRSRR at each resonance frequency. This visualization helps us understand the current flow through the brass zipper and CRSRR and the impact of their capacitive separation. It also demonstrates the parasitic current sharing between each pair of CRSRRs, with the largest one serving as the primary conductor. Additionally, it reveals that adding CRSRR cells can suppress the surface waves, thereby enhancing the radiation characteristics of LWTA. The CRSRR cells follow five periodic growth factors. Each ring acts as a capacitor, functioning in parallel with a resistor and inductor connected in series. Furthermore, a coupling capacitor facilitates communication between the two rings.

**Fig. 11:**
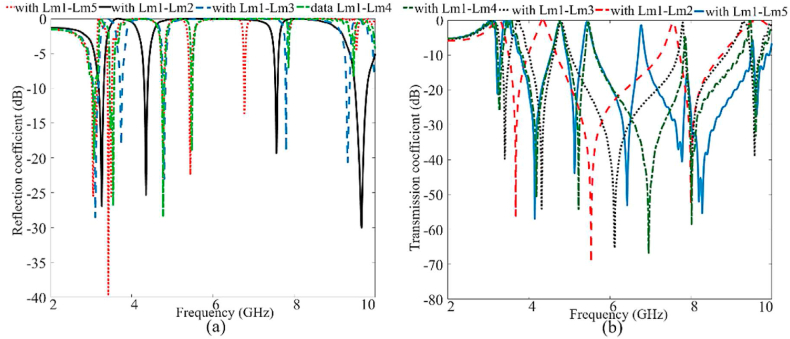
The S-parameters and extracted permittivity and permeability results of the CRSRR integrated with brass zipper.

Permittivity and permeability are characteristics of the metamaterial (MTM) structure, indicating its ability to enhance the LWTA's characteristics at particular frequencies. Fig. 11 illustrates the results of the S-parameters for the individual rings when integrated with the brass zipper and when all rings are interconnected at the end. The resonances associated with each split ring become apparent when all five CRSRRs are utilized. Furthermore, the inclusion of MIMO LWA effectively eliminates unwanted resonance. It is combined with the proposed MIMO LWTA to improve the antenna's directional gain, beam scanning capability, and radiation in an outward direction. Additionally, the overall performance of the MIMO LWA is investigated, and it remains relatively stable when the zipper is opened at different angles. The optimized specific parameters of the MTM structure can be found in Table 1.

**Fig. 12:**
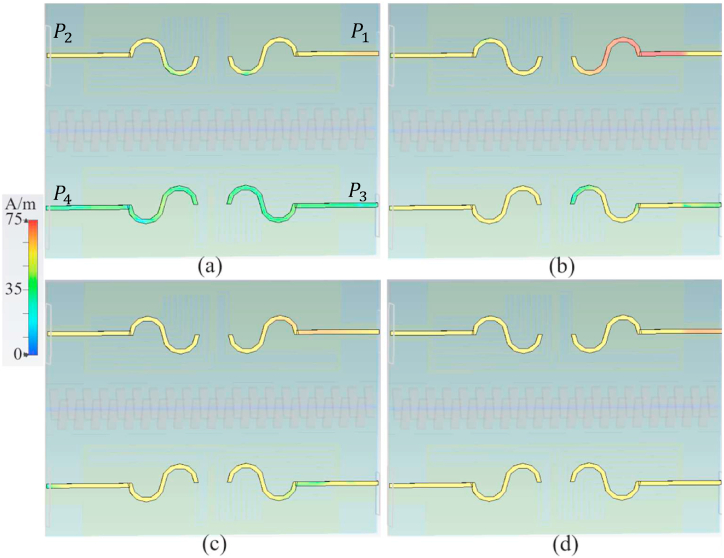
The SCD of the S-shaped neutralization network at a: 3.4 GHz, b: 5.6 GHz, c: 7.2 GHz, and d: 8.5 GHz.

After evaluating the performance of the MIMO LWTA equipped with the brass zipper and nested CRSRR MTM cells, the material of the zipper was changed to APP, resulting in corresponding alterations to the neutralization network (it is to investigate both materials that are usually used to make zippers in the market). The neutralization network, integrated with the APP zipper, comprises four S-shaped resonators and transmission lines starting from the antenna's edge. To ensure the desired resonance and understand the impact of the transmission line's length and width on antenna performance, an examination of the surface current on the second layer is necessary. Fig. 12 illustrates the SCD of the MIMO LWTA with APP zippers and the layer of the neutralization network containing the S-shaped patches (port 1 (P1) is active). As shown in Fig. 12, when port one is active, the surface current is not prominently distributed at higher frequencies around ports three and four. It exhibits greater strength around the transmission line at 3.4 GHz, followed by a slight decrease. Furthermore, two shorting pins (Fig. 8) are added at the junction of the transmission lines and S-shaped patches at ports one and 3. These shorting pins, positioned closer to the edges and ports, enhance the antenna's resistance to bending and crumpling. These pins are used to reduce the surface waves around the patches and also to improve the isolation since the APP zipper cannot induct any current. The inclusion of the S-shape patches was motivated by their ability to provide multi-band functionality, impedance-matching improvement, and anti-reflection properties. These patches were created by connecting two halves of circular rings. The optimization process of the proposed MIMO LWTA involves fine-tuning each section and design parameter to ensure that the desired values are achieved specifically at the resonances.

## 3 Result and discussion

**Fig. 13:**
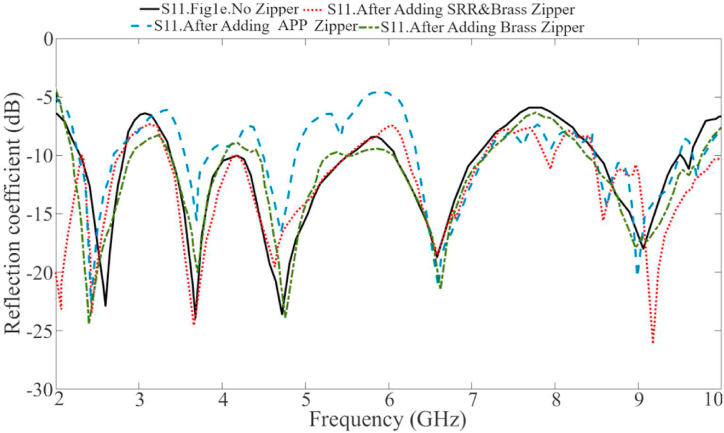
The reflection coefficient results of the antenna at a different stage of the design with the APP and Brass zipper.

The performance of the antenna on and off the body is presented in this section. Initially, we examine the antenna when not in contact with the body (in the air). Then, the antenna's performance is examined when it is being worn. Fig. 13 illustrates the reflection coefficients obtained from integrating MIMO LWTA with and without the APP zipper, the S-shape neutralization network, and the implementation of T and L-shaped slots. The APP zipper's presence significantly enhanced the antenna's performance, particularly in impedance matching. This improvement can be attributed to the APP zipper's dielectric resonator role, effectively reducing surface waves and inductive loss. The positive impact of the APP zipper on the reflection coefficient and the enhancement of the bandwidth can be observed in Fig. 13.

The combination of the five rectangular rings and the brass zipper generates five resonances, enhancing the level of matching and reflection coefficients upon integration with the LWA. This integration acts as an inductive loading mechanism, causing the resonances to shift to lower frequency ranges. Consequently, the CRSRR, second layer, and brass zipper are merged with the LWA MIMO, resulting in improved performance compared to adding the S-shape network. Furthermore, this integration successfully achieves the desired frequencies of 1.7–2.8 GHz, 3.45–4.1 GHz, 4.2–5.5 GHz, 6.5–7.3 GHz, and 8.7–9.85 GHz when combining the brass zipper with the MIMO LWTA. The antenna integrated with the brass zipper has more working bands, especially when fully closed and all the CRSRRs are connected. A few of these bands are no longer available when the brass zipper is opened. However, the desired bands are still intact and work.

### 3.1 Off-body investigation results

**Fig. 14:**
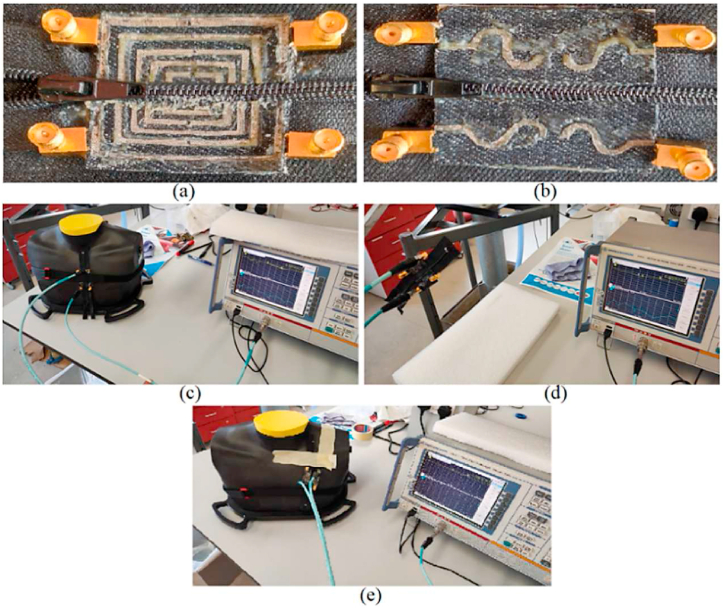
The measurement setup of the proposed antenna in both air and on-body (a, b: fabricated zipper antennas; c, e: measurement on chest; d: measurement in free space).

Fig. 14 depicts the fabricated models developed for validating the theory. Denim and Shieldit Super Fabric conductive textiles were utilized to construct the antenna model. The layers have adhered together using a plastics-to-textiles adhesive. The antenna's S-parameters were measured using a two-terminal HP 8510C vector network analyzer (VNA). Fig. 14 also depicts the fabricated proposed antennas along with the measurement setup. The measurement process involved three stages: initially connecting Ports 1 and 2, followed by Ports 3 and 4. Then, measuring free space and on a human phantom/body are performed.

**Fig. 15:**
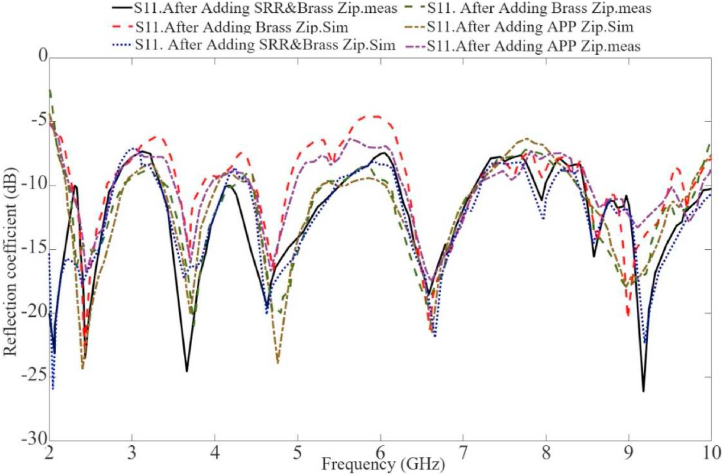
The antenna's reflection coefficient results in the air (off-body) (‘sim’ is a simulation, 'meas' is measurement).

**Fig. 16:**
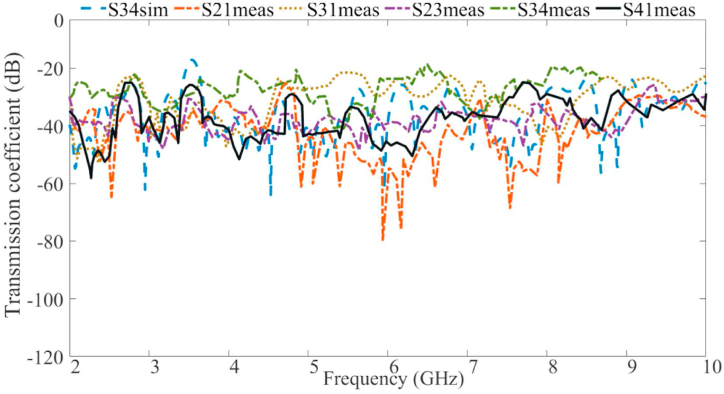
The antenna's reflection coefficient results in the air (off-body) (‘sim’ is a simulation, 'meas' is measurement).

A comparison between the simulated and measured S-parameters is presented in Fig. 15, Fig. 16. The simulated and actual results demonstrate a good agreement, although some slight irregularities are observed in the upper band due to fabrication tolerances. However, the antenna still functions as intended when utilized within the desired bands and the applications that were the purposes of this paper. In addition to the S-parameters measurement, the antenna's radiation pattern was also obtained by utilizing a VNA and an anechoic chamber. This analysis took place within the frequency range of 0.1–10 GHz. The procedure involved measuring and assessing the S-parameter data to compare with the equivalent modeling data. Antennas were used to conduct the measurements, one connected to the first terminal of the VNA and the other connected to the second terminal. In the context of radiation pattern measurement, a reference horn antenna is affixed to a rotating rod attached to a power meter for assessing the antenna's radiation pattern. The measurement setup includes a chamber with a movable vertical wall on the left side, likely for repositioning purposes. Shifting the wall enables the antenna to be adjusted for measurements on both sides of the chamber, facilitating a comprehensive evaluation of the antenna's radiation pattern and how its signal propagates in different directions. An alternative approach involves attaching the recommended antenna to a pole segment and incrementally rotating it by 3° using a motion controller. Data is recorded after each rotation, and MATLAB generates the radiation pattern. The process also involves rotating the antenna into the elevation plane to complete the measurement process.

**Fig. 17:**
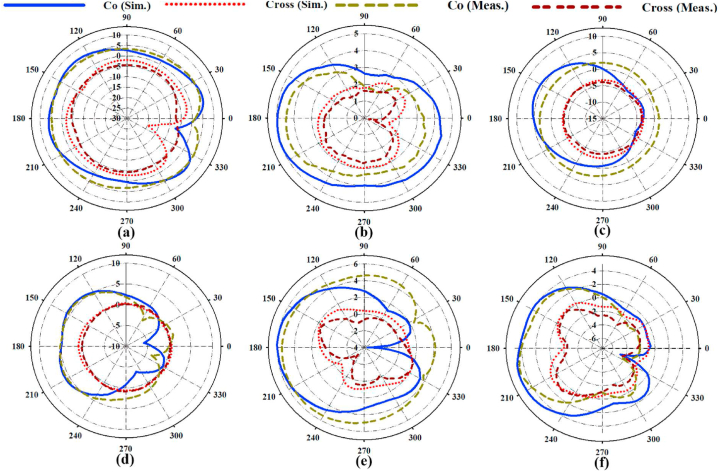
The radiation pattern of the antenna (simulated (sim) and measured (meas) cross and co-polarization) at (a) 3.2 GHz, (b) 3.4 GHz, (c) 4.2 GHz, (d) 5.6 GHz, € 8.25 GHz, and (f) 9.25 GHz.

Fig. 17 illustrates the measured and simulated electrical (E) (XY-plane) and magnetic (H) (XZ-plane) far-field radiation patterns for the antenna. The variation between the simulated and experimental results was negligible (discrepancies in fabrication tolerances throughout the manufacturing process may explain the slight variation in outcomes). Peak gain for all ports ranged from 5 to 12 dBi for co-polarization and from −8 to −10 dBi for cross-polarization, indicating that the antenna's radiated power is focused in the desired direction. However, some energy is also radiated in other directions. In a multi-path scenario, this value of cross-polarization is not a fundamental matter of concern. Moreover, the antenna's radiation pattern is entirely external to the body, with the primary lobe pointing towards approximately 180° (directed outward from the patch in the Z direction), making it a promising choice for wearable antenna systems and communication.

The ability to steer the beam is a crucial aspect of LWAs. The beam needs to have a wide steering range to achieve more coverage. The beam scanning capability of the LWA at different frequencies and for each design stage was examined. The steering range of the antenna with and without loading, with zippers and second layers, was also evaluated. The antenna with an APP zipper provides full-space steering coverage (beyond 160°) for all working bands. The antenna with brass zipper offers a broader range of steering angle (180°) at all the working bandwidth nearly in both elevation and azimuth planes.

### 3.2 On-body investigation results

**Fig. 18:**
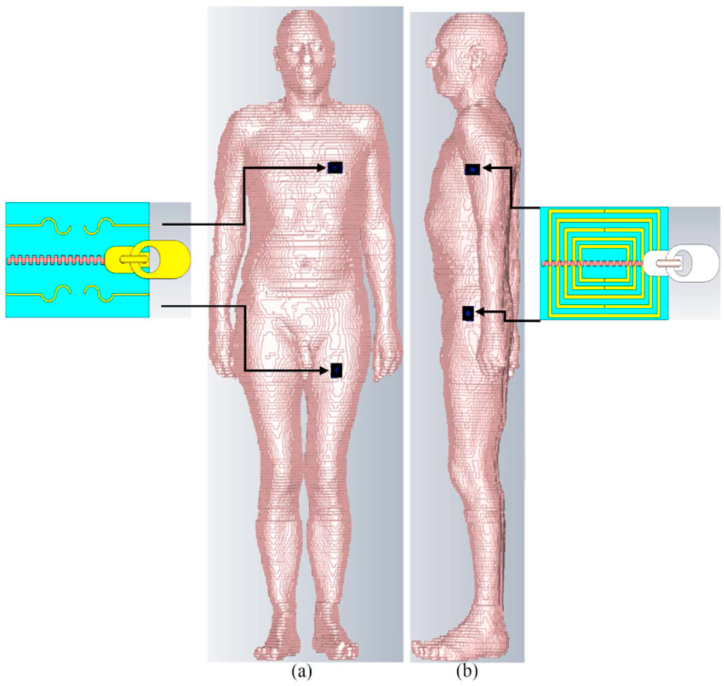
The proposed antenna's simulation setup is on the chest, arm, and limb (a: the APP zipper antenna on the limb and chest, b: the brass zipper antenna on the limb and arm).

In this subsection, we examine the proposed antenna's performance when placed on the body in terms of S-parameters, radiation pattern, gain, efficiency, axial ratio (AR), and SAR value. Fig. 18 illustrates the simulated configuration for the on-body conditions, including the voxel arm, chest, and limb. The separation gap between the antenna and the voxel body is less than 5 mm.

**Fig. 19:**
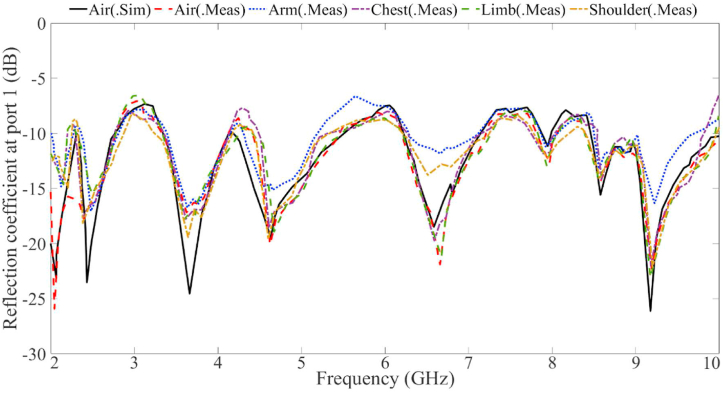
The reflection coefficient results of the proposed antenna with CSRRs and brass zipper on the voxel body at port 1.

**Fig. 20:**
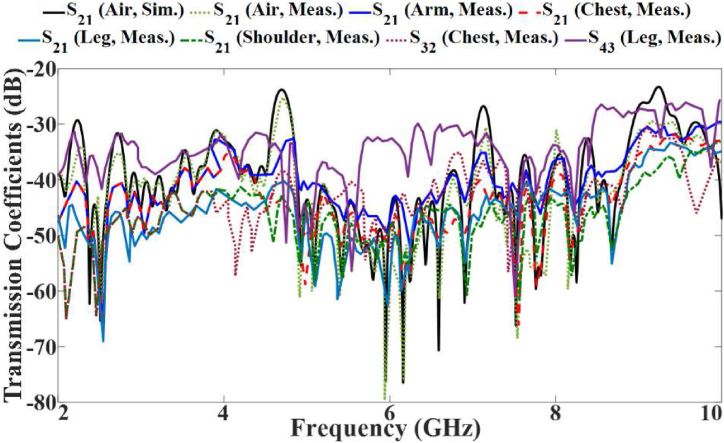
The transmission coefficient results for the proposed antenna with a brass zipper on the voxel body.

The simulated S-parameter results for the voxel body scenarios are presented in Fig. 19, Fig. 20. These findings demonstrate that all the desired frequencies and bandwidths are available and functional. However, physiological characteristics of the body cause slight shifts in the bands on the voxel. Additionally, the voxel results exhibit more band notches than the results in free space. Nevertheless, the required bands perform as expected within the system. The transmission coefficient between any two ports remains below −20 dB across the entire useable bandwidth, ensuring excellent port isolation.

**Fig. 21:**
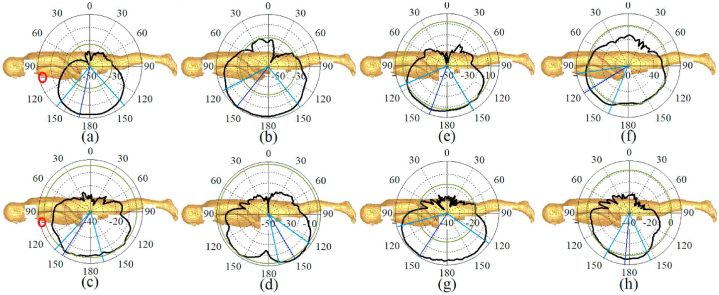
The simulated radiation pattern of the antenna on the chest for APP zipper (a–d) and brass zipper (e–h) at a,e: 3.4 GHz; b,f: 5.6 GHz; c,g: 4.2 GHz; d,h: 8.25 GHz (The red circle is the location of the antenna).

**Fig. 22:**
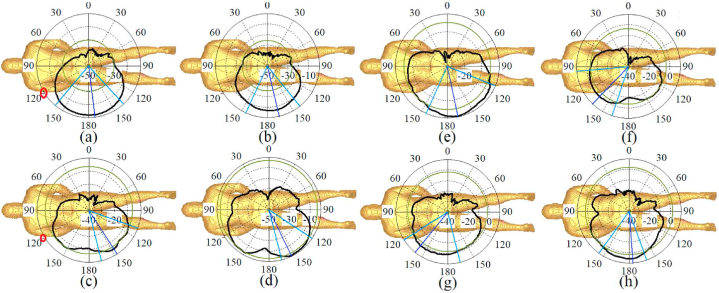
The simulated radiation pattern of the antenna on the arm for APP zipper (a–d) and brass zipper (e–h) at a,e: 3.4 GHz; b,f: 5.6 GHz; c,g: 4.2 GHz; d,h: 8.25 GHz (the red circle is the location of the antenna).

**Fig. 23:**
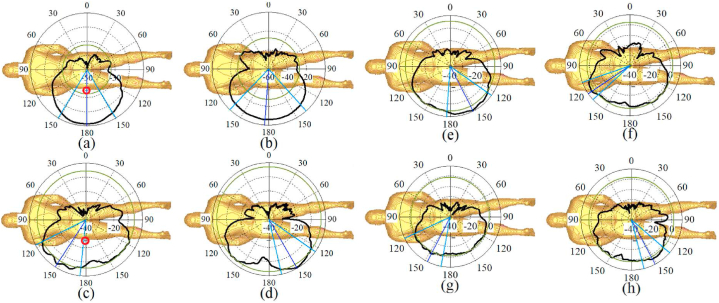
The simulated radiation pattern of the antenna on a limb for APP zipper (a–d) and brass zipper (e–h) at a,e: 3.4 GHz; b,f: 5.6 GHz; c,g: 4.2 GHz; d,h: 8.25 GHz (the red circle is the location of the antenna).

Fig. 21, Fig. 22, Fig. 23 illustrate the antenna's radiation pattern when placed on different areas of the human body. These areas are the chest, arm, and limb. When mounted on the voxel body, the antenna emits high gain radiation in all directions except inward of the body for both types of zippers. At lower frequencies, the radiation is concentrated outward of the user's chest, while at higher frequencies, it shifts within the range of 30° (as shown in Fig. 21). The radiation from the arm and limb also points outward, with the majority falling within the range of 150–180° for the arm and around 150–170° for the limb. Furthermore, the radiation patterns remain consistent across higher and lower frequency bands, consistently directing the radiation away from the body for both neutralization networks.

**Fig. 24:**
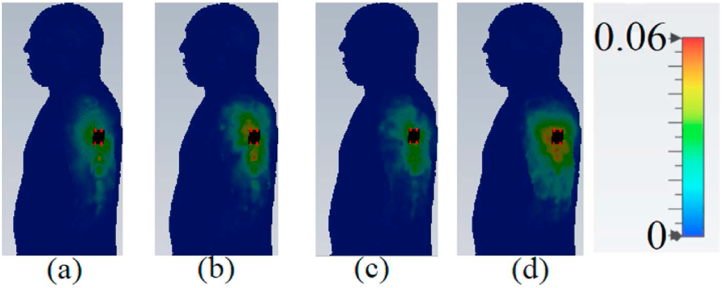
The proposed antenna's SAR evaluation on the arm (a: 3.4 GHz, 1 g; b: 4.2 GHz, 1 g; c: 5.6 GHz, 1 g; and d: 8.25 GHz, 1 g).

**Fig. 25:**
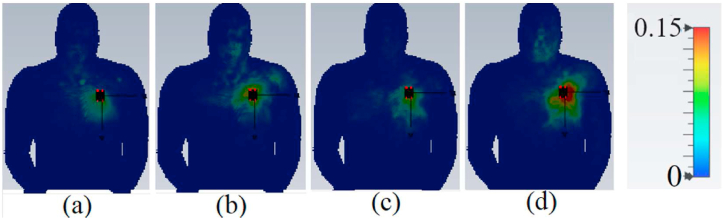
The proposed antenna's SAR evaluation on the chest (a: 3.4 GHz, 1 g; b: 4.2 GHz, 1 g; c: 5.6 GHz, 1 g; and d: 8.25 GHz, 1 g).

**Fig. 26:**
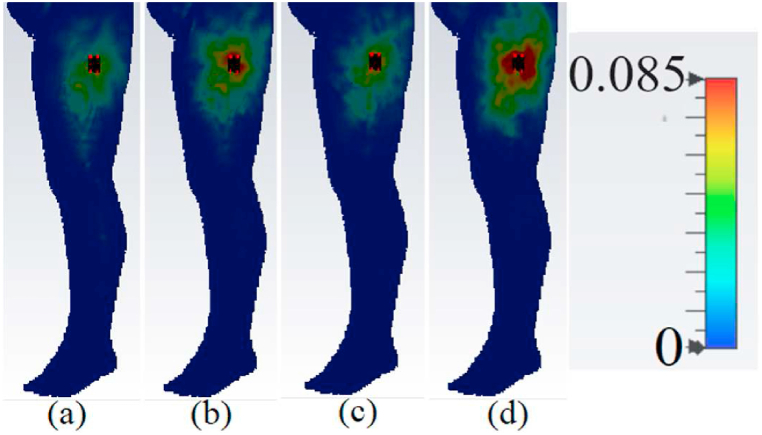
The proposed antenna's SAR evaluation on a limb (a: 3.4 GHz, 1 g; b: 4.2 GHz, 1 g; c: 4.2 GHz, 1 g; and d: 8.25 GHz, 1 g).

When evaluating the performance and radiation of a wearable antenna, it is crucial to consider the SAR. The SAR values should adhere to the guidelines set by American and European standards, which specify that the SAR values should be below 2 and 1 W/kg3 [23]. The impact of SAR variation on voxel human tissue (such as the arm, chest, and limb) is depicted in Fig. 24, Fig. 25, Fig. 26. Simulated SAR values can be found in Table 2, confirming that the SAR values are well within the safe range. This indicates that the presence of the antenna does not significantly affect the human voxel, with a gap of 5 mm and 8 mm between the antenna and voxel.

**Table 2:** The comparison of the brass zipper antenna's performance in free space, arm, chest, and limb.

f_r_ (GHz)		3.4	5.6	8.25
Free space	Gain (dBi)	12.15/10.55	10.8/9.91	7.24/6.93
	Sim/Meas			
	Eff (%)	95.5	92.4	89.6
On chest	Gain (dBi)	11.00/9.95	8.94/7.45	7.65/6.23
	Sim/Meas			
	Eff (%)	91.41	84.12	89.14
	SAR (1 g/10 g)	0.024/0.010	0.033/0.017	0.120/0.042
	(W/kg)			
On arm	Gain (dBi)	8.35/-	7.14/-	5.95/-
	Sim/Meas			
	Eff (%)	84.7	93.23	83.31
	SAR (1 g/10 g)	0.053/0.017	0.052/0.017	0.040/0.020
	(W/kg)			
On limb	Gain (dBi)	7.05/-	7.09/-	6.10/-
	Sim/Meas			
	Eff (%)	84.3	91.33	83.45
	SAR (1 g/10 g)	0.081/0.009	0.038/0.018	0.076/0.035
	(W/kg)			

**Fig. 27:**
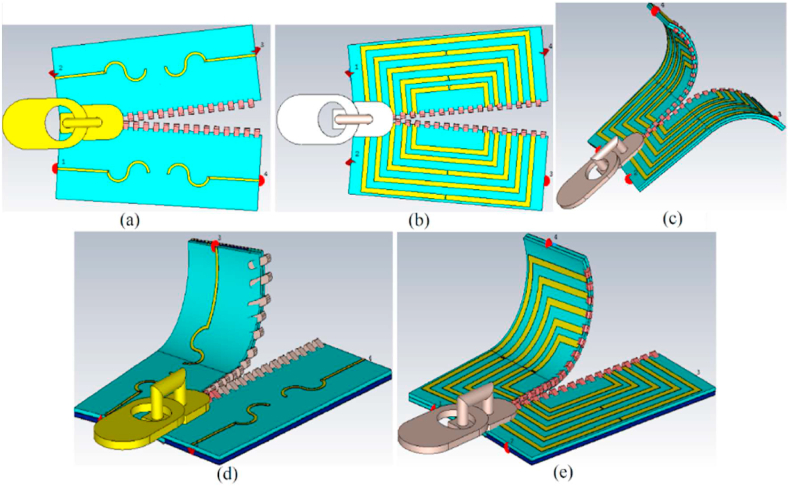
The flexibility evaluation setup towards the X and Y axis (a: 30 °, b: 30 °, c: 90 °, d: 110 °, e:110 °).

**Fig. 28:**
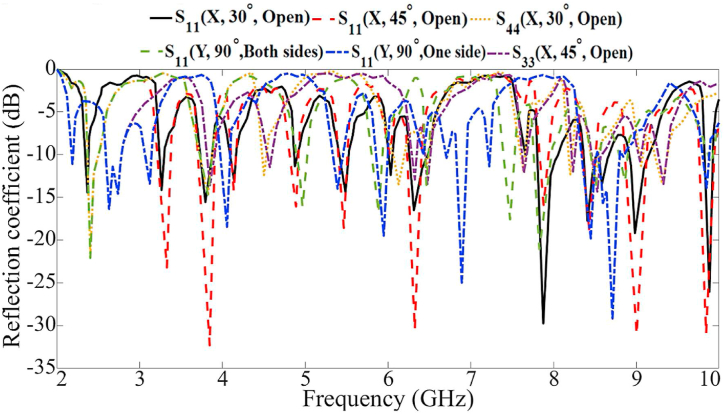
The antenna's reflection coefficient results during flexibility evaluation.

**Fig. 29:**
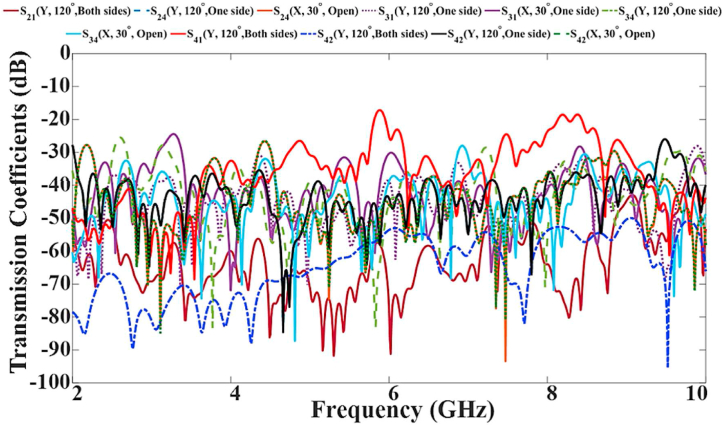
The antenna's transmission coefficient results during flexibility evaluation.

The ability of the antenna to resist folding is then assessed. We tested various scenarios to simulate real-world wearable conditions and evaluate the antenna's performance. These included folding the antenna, opening the zippers up to a 40-degree angle, and folding the LWA elements independently along the X and Y axes. These tests were conducted to observe the antenna's functionality after being worn. The antenna strength was evaluated separately along the X and Y axes, as shown in Fig. 27(a)–(e). Fig. 28, Fig. 29 demonstrate that the LWTA can withstanding twisting up to 175° in both the X and Y directions while maintaining the desired performance.

The variation of the S-parameter of the proposed MIMO LWTA under the flexibility evaluations is shown in Fig. 28, Fig. 29. They show a slight shift in the antenna's reflection and transmission coefficients because of the folding. As a result, several stopbands were created in the operational bandwidth (BW) after bending. However, the band still includes the resonances critical for applications such as WBAN and communication bands of L, C, S, and X bands. They also depict how the antenna's transmission and reflection coefficients shift after a rotation. In the functional BW, several stopbands were formed after stretching. Despite this, the device keeps functioning successfully in those bands.

**Fig. 30:**
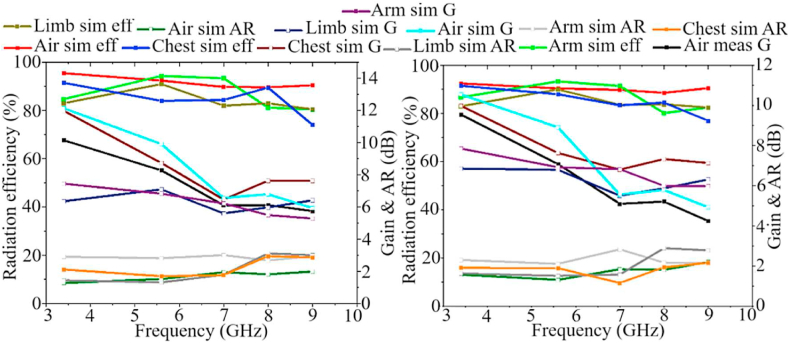
Gain, AR, and radiation efficiency results of MIMO antenna in air and on the body (a: with brass zipper and b: with APP zipper).

**Fig. 31:**
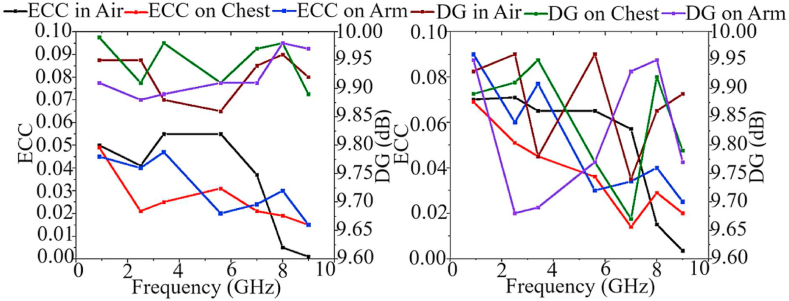
DG and ECC values of MIMO antenna in air and on the body (a: brass zipper and b: APP zipper).

After the antenna's agility is demonstrated by exploring and analyzing three factors in both on-body and off-body settings, the diversity ability of the MIMO antenna is assessed. The diversity evaluation of the MIMO antenna is depicted in Fig. 30, Fig. 31. The DG of ten and the ECC of less than 0.05 highlight the excellent diversity the suggested MIMO antenna provides. Moreover, the AR remains below three. It is important to note that the values of these parameters are likely to be almost consistent in both on-body and off-body scenarios. Additionally, the effects of these parameters were minimized when the antenna was close to the skin.

Table 3 provides a comparison between the proposed work and recently developed multiband four-port MIMO antennas tailored for wearable applications, including 5G and WBANs. The comparison encompasses various essential factors for wearable MIMO antennas, such as the number of operating bands, maximum gain, radiation efficiency, dimensions, DG, and ECC. For instance Ref. [24], introduced a versatile UWB textile antenna with notch characteristics, covering frequencies from 3.18 to 4.60 GHz and 8.89–12 GHz, achieving isolation between ports of around 20.5 dB. However, despite its wide frequency coverage, it had relatively large physical dimensions. Another example is a flexible MIMO antenna designed for Sub-6 GHz 5G and X-band frequencies, with a compact structure covering bandwidths from 3.34 GHz to 5.01 GHz (40 %) and 8.9 GHz–9.2 GHz (3.31 %). It achieved a minimum efficiency of 70 %, a maximum gain of 4 dBi, and an ECC below 0.17 [25]. Other wearable antennas, while achieving a bandwidth of 0.6 GHz and a maximum gain of less than four dBi with a size of 88.7 × 68.7 mm^2^ [15], did not offer high gain or multiband features despite their larger dimensions and failed to consider most MIMO diversity features. On the other hand, works presented in Refs. [17,23,[26], [27], [28]] considered more MIMO diversity characteristics like DG and ECC. Though all were wearables, not all assessed the possibility of polarization except for [26,27,29]. [23,26] offered UWB antennas covering multiple bands, but their gains were too low even with large sizes. Notably [29,30], considered all parameters, offering wideband and multiband features, maximum gains up to 7.1 dBi, diversity gains of less than 10 dB, and ECC of 0.002 and 0.0001, respectively. Despite their notable achievements, the proposed antenna demonstrates superior performance regarding the number of working bands, gain, dimensions, radiation efficiency, DG, and ECC.

**Table 3:** Comparison of the proposed antenna integrated with brass zipper with previously reported work on MIMO antennas (four-port/wearables).

References	Freq. (GHz) Off/On	Polarization	Max Eff (%)	Max Gain (dBi)	Dim (mm^2^) L × W	DG	Ports/wearable	ECC
[15]	4.4–5	N/A	90	<4	88.7 × 68.7	N/A	4/yes	N/A
[17]	2.74 to 4.41	N/A	N/A	<5	58×55	>9.6	4/yes	<0.2
[23]	2.4–2.484, 3.2–3.85, 5.15–5.35, 5.72–5.825	N/A	93	N/A	55 × 35	<9.97	2/yes	<0.06
[24]	3.18–4.60, 8.89–12	N/A	N/A	<6	40 × 40	N/A	4/yes	<0.2
[25]	3.34–5.01, 8.9–9.2	N/A	N/A	N/A	54.97 × 54.97	N/A	4/yes	<0.17
[26]	2.75–12	LP	89.3	3.41	4 (10 × 40)	>9.5	4/yes	<0.18
[27]	5.0–6.6	CP	83	10	60 × 44	>9.7	4/yes	<0.05
[28]	4.36–6.9	N/A	90	2	25 × 25	>9.9	4/no	<0.08
[29]	3–5	CP	>95	7.1	63 × 63	>9.9	4/no	<0.002
[30]	2.2–3.5	N/A	<97	<6	60 × 60	<10	4/yes	0.0001
	4.8–6.2							
	7.8–9.8							
**This work**	1.7–2.8, 3.45–4.1, 4.2–5.5, 6.5–7.3,	CP	95.5	12.15	40 × 30	<10	4/Yes	<0.05
	8.7–9.85							

## 4 Conclusion

A four-port MIMO LWTA antenna integrated with zippers is proposed to operate across multiple frequencies. This innovative design combines two different zipper materials, an LWA, and two neutralization networks for each zipper. It is also designed to achieve wearer comfort, increased diversity, channel capacity, and data rate transmission. The antenna covers the frequency bands necessary for WBANs, 5G, sub-6 GHz, and X-band communications, specifically ranging from 1.7 to 2.8 GHz, 3.45–4.1 GHz, 4.2–5.5 GHz, 6.5–7.3 GHz, and 8.7–9.85 GHz. It offers a maximum radiation efficiency of 95 % and a gain of 12.15 dBi, exhibiting CP.

The integration of the zippers with two different materials, the combination of brass zipper and the CRSRR, the S-shape patches layer and APP zipper, and the LWA MIMO results in a wider BW, increased efficiency, gain, dual-polarization (DP), and compact dimensions compared to recent research in this field. The LWA MIMO's dual polarization, low ECC below 0.05, nearly 10 dB DG, AR below 3, and maximum isolation of 23 dB contribute to its successful application as a MIMO antenna with a high degree of diversity.

Extensive simulations and measurements were conducted using voxel-body models to assess the antenna's performance in both off-body and on-body scenarios. Folding and tilting analyses and open zippers with different angles were also performed to evaluate the proposed antenna's adaptability. The antenna demonstrates promising results, exhibiting a strong correlation between simulated and measured outcomes. Moreover, MIMO performance characteristics such as ECC and DG confirm that the proposed antenna is well-suited for MIMO systems.

The proposed antenna is potentially a solution for various communication applications, including laptop communications, air traffic, and defense tracking, facilitating better coordination among ground-based soldiers and nurses. It also emerges as a reliable candidate for WBAN applications and 5G communications due to its impressive performance and adaptability.

## CRediT authorship contribution statement

**Tale Saeidi:** Writing – original draft, Validation, Software, Methodology, Formal analysis, Conceptualization. **Sahar Saleh:** Investigation, Formal analysis, Data curation. **Sarmad Nozad Mahmood:** Software, Investigation. **Nick Timmons:** Writing – review & editing, Validation, Supervision, Project administration, Funding acquisition, Formal analysis, Data curation. **Ahmed Jamal Abdullah Al-Gburi:** Software, Investigation, Formal analysis, Data curation. **Saeid Karamzadeh:** Writing – review & editing, Validation, Supervision, Methodology, Data curation. **Faroq Razzaz:** Writing – review & editing, Validation, Methodology, Funding acquisition, Formal analysis, Data curation.

## Declaration of competing interest

The authors declare the following financial interests/personal relationships which may be considered as potential competing interests:Tale Saeidi reports was provided by Atlantic Technological University. Dr. Nick Timmons reports a relationship with Atlantic Technological University that includes: board membership, employment, and funding grants. None has patent none with royalties paid to none. There is no conflict of interest among the authors of this article.
